# Enhancement of catecholamine release from PC12 cells by the traditional Japanese medicine, rikkunshito

**DOI:** 10.1186/1472-6882-14-256

**Published:** 2014-07-19

**Authors:** Yuko Nagamura, Kiyoshi Terawaki, Yasuhito Uezono, Toshihiko Tsukada

**Affiliations:** 1 Division of Familial Cancer Research, National Cancer Center Research Institute, 5-1-1 Tsukiji, Chuo-ku, Tokyo 104-0045, Japan; 2 Division of Cancer Pathophysiology, National Cancer Center Research Institute, 5-1-1 Tsukiji, Chuo-ku, Tokyo 104-0045, Japan; 3 Present address: JBIC Research Institute, Advanced Industrial Science and Technology, 2-4-7 Aomi, Koto-ku, Tokyo, Japan; 4 Present address: Tsumura Research Laboratories, Tsumura & Co, 3586 Yoshiwara, Ami-machi, Inashiki-gun, Ibaraki 300-1192, Japan

**Keywords:** Rikkunshito, Catecholamine, PC12, cAMP

## Abstract

**Background:**

Rikkunshito is a traditional Japanese herbal medicine that is used to treat appetite loss associated with cancer and other disorders. The formulation contains various constituents that influence cell signaling, and rikkunshito may accordingly affect human homeostasis through multiple regulatory pathways, including those governed by the endocrine system. We investigated the actions of rikkunshito on catecholamine release from PC12 cells, an adrenal chromaffin cell line.

**Methods:**

The actions of rikkunshito on PC12 cells were evaluated by measuring intracellular cAMP levels, tyrosine hydroxylase (TH) and vasoactive intestinal peptide (VIP) mRNA expression levels, and catecholamine levels in the culture medium. The transcriptional activation of VIP gene by rikkunshito was assessed by using a VIP promoter-driven reporter gene assay.

**Results:**

Rikkunshito dose-dependently enhanced forskolin-induced elevations in cAMP in PC12 cells, and also increased the gene expression of TH and VIP. The transcriptional activation of VIP gene by rikkunshito was confirmed. Norepinephrine and dopamine secretion into the culture medium of PC12 cells were also dose-dependently augmented by rikkunshito and/or forskolin, but experiments with a protein kinase C (PKC) activator and a phosphodiesterase inhibitor revealed that the effects of rikkunshito were not simply due to the modulation of PKC or phosphodiesterase activity.

**Conclusions:**

These findings suggest that rikkunshito enhances the release of catecholamines by a novel mechanism involving cAMP.

## Background

Rikkunshito is a traditional Japanese herbal medicine that is used to treat various symptoms of dyspepsia, including appetite loss, nausea, vomiting, and epigastric distention [1-4]. Rikkunshito is not commonly associated with serious adverse effects; hence, this formulation has recently received attention for the management of cancer-related conditions, such as cancer cachexia [5-8]. Rikkunshito (Japan standard commodity classification No. 875200) consists of eight crude drugs: *Atractylodis lanceae rhizoma* (atractylodes lancea rhizome, 18.6% (w/w)), *Poria* (poria sclerotium, 18.6%), *Ginseng radix* (ginseng, 18.6%), *Pinelliae tuber* (pinellia tuber, 18.6%)*, Aurantii nobilis pericarpium* (citrus unshiu peel, 9.3%), *Zizyphi fructus* (jujube, 9.3%), *Zingiberis rhizoma* (ginger, 2.3%), and *Glycyrrhizae radix* (glycyrrhiza, 4.7%). Because many of these herbal constituents influence cell signaling, rikkunshito may affect human homeostasis through multiple regulatory pathways, including the endocrine system.

PC12 cells are derived from a pheochromocytoma of the rat adrenal medulla and display the characteristics of adrenal chromaffin cells. PC12 cells are therefore often used as a model of the adrenal medulla in pharmacological studies [9]. These cells contain endogenous catecholamines that are released in response to distinct stimuli, including various herbal medicine components. For example, *Salviae miltiorrhizae* radix, a Korean herbal medicine, increases dopamine release from PC12 cells [10]. On the other hand, hirsuteine, a compound isolated from a Japanese herb of the *Uncaria* genus, antagonizes the nicotine-evoked secretion of dopamine [11]. In addition, extracts of ginseng, a component of rikkunshito, suppress the gene expression of enzymes involved in dopamine and norepinephrine synthesis in PC12 cells [12]. However, in spite of extensive studies utilizing PC12 cells, the effects of rikkunshito itself on the synthesis and/or release of catecholamines have never before been documented. We have been interested in the influences of Japanese herbal medicine on the endocrine system and examined the effects of rikkunshito on endocrine cells. We now report that rikkunshito stimulates the release of norepinephrine and dopamine from PC12 cells via a novel mechanism involving cAMP.

## Methods

### Rikkunshito stock solution

Rikkunshito was kindly supplied by Tsumura and Co. (Tokyo, Japan) as a spray-dried powder derived from a hot water extract of crude drugs. The spray-dried powder (10 g) was resuspended in hot water (100 ml), and the supernatant was filtered through a 0.45 μm sterile membrane (Millipore, Ireland). Phosphate buffered saline (10× stock solution, 1:9 v/v) was added to the filtrate to prepare the rikkunshito stock solution. Aliquots of the rikkunshito stock solution were stored at -30°C until use.

### Cells

PC12-VG cells were previously established by the stable transfection of the β-galactosidase reporter gene transcribed from the vasoactive intestinal peptide (VIP) gene promoter into the parental rat pheochromocytoma PC12 cell line [13]. Cells were maintained in Dulbecco’s modified Eagle’s medium supplemented with 10% fetal bovine serum (ICN Biomedicals, Inc., Aurora, OH), 5% horse serum (Gibco, Life Technologies, Carlsbad, CA), penicillin-streptomycin (100 U/ml penicillin and 100 μg/ml streptomycin) (nacalai tesque, Kyoto, Japan), and 100 μg/ml G418 disulfate aqueous solution (nacalai tesque), as previously described [13].

### Measurement of intracellular cAMP levels

Semi-confluent cells plated in 24-well plates were treated in triplicate for 30 min with forskolin (0, 0.1, 0.3, 1, or 3 μM) (Calbiochem, Merck, Damstadt, Germany) plus rikkunshito stock solution (0, 1, or 3%). Cells were then subjected to a competition enzyme-linked immunoassay by using the cAMP XP™ assay kit (Cell Signaling Technology, Danvers, MA).

### Measurement of mRNA expression levels

Semi-confluent cells plated in 60 mm tissue culture dishes were treated in triplicate for 6 h with forskolin (0, 0.1, 0.3, 1, or 3 μM) plus rikkunshito stock solution (0 or 3%). Total RNA was extracted by using the QIAshredder (Qiagen, Hilden, Germany) and the RNeasy Mini Kit (Qiagen). First strand cDNA was prepared by using oligo-dT primers and SuperScript III reverse transcriptase (Invitrogen, Life Technologies). Rat tyrosine hydroxylase (TH) and rat VIP mRNA levels were measured by using the QuantiTect SYBR Green RT-PCR assay kit (Qiagen) and a real-time monitoring fluorescent quantitative detection system (LineGene, Bioflux, Tokyo, Japan). The mRNA levels for each gene were normalized relative to those of glyceraldehyde-3-phosphate dehydrogenase (G3PDH). The primer sequences were as follows: TH, forward 5′-AGCCC AAGGG CTTCA GAAGG GC-3′, reverse 5′-CGCTC CTTGC GGGCA TCCTC GATGA G-3′; VIP, forward 5′-CGCTG GCCTG GCCTC TCTAT G-3′, reverse 5-CTGCA AGATG TCAGA GTCTG C-3′; and G3PDH, forward 5′-TGCAC CACCA ACTGC TTAGC-3′, reverse 5′-AGTGA TGGCA TGGAC TGTGG-3′.

### Measurement of VIP gene promoter activity

Semi-confluent cells plated in a flat-bottomed 96-well plate were treated in quadruplicate for 6 h with fresh culture medium containing forskolin (0, 0.1, 0.3, 1, 3, or 10 μM) plus rikkunshito stock solution (0, 1, or 3%), 100 nM 12-O-tetradecanoylphorbol-13-acetate (TPA) (Wako Pure Chemical Industries, Osaka, Japan), or 0.5 mM 3-isobutyl-1-methylxanthine (IBMX) (Sigma-Aldrich, St. Louis, MO). VIP gene promoter activity was determined by assaying β-galactosidase activity with a chromogenic substrate, measuring the absorbance at 420 nm, and subtracting the background absorbance, as described previously [13].

### Measurement of catecholamines in culture medium

Cells were treated in triplicate with forskolin (0, 0.1, or 0.3 μM) plus rikkunshito stock solution (0, 1, or 3%), 100 nM TPA, or 0.5 mM IBMX. Three hours later, aliquots of the culture medium were collected and stored at -20°C until use. Catecholamine levels were analyzed by high performance liquid chromatography.

### Statistical analysis

Statistical analyses of mean values in the treatment vs. control at each forskolin concentration were performed by two-tailed Student’s *t*-test. Data were expressed as the mean ± standard error of the mean (SEM), and *P* values less than 0.05 were considered statistically significant.

## Results

### Effects of rikkunshito on cAMP levels in PC12 cells

**Figure 1:**
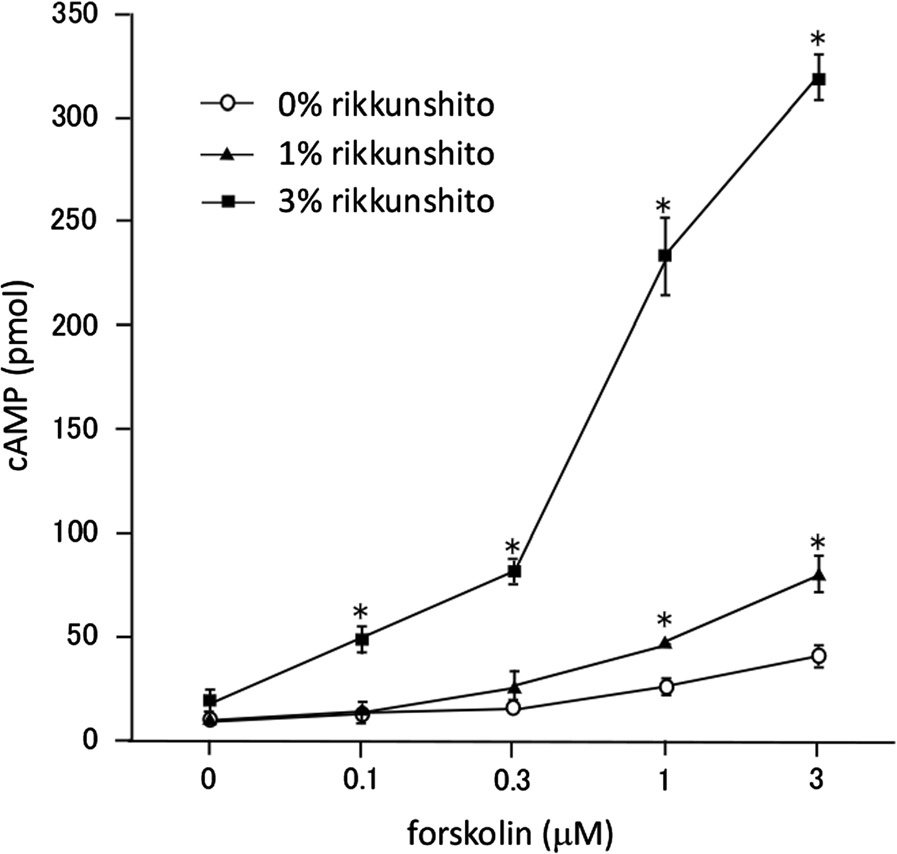
**Rikkunshito acts in synergy with forskolin to enhance cAMP levels in PC12 cells.** PC12 cells were treated with forskolin alone or in combination with rikkunshito for 30 min. cAMP levels are shown as total amounts for cells plated in individual wells of 24-well plates. Data are presented as the mean ± SEM of triplicate independent experiments. *, p < 0.05 vs. 0% rikkunshito at each forskolin concentration tested, two-tailed Student’s *t*-test.

Forskolin, a direct activator of adenylate cyclase, dose-dependently increased intracellular cAMP levels in PC12 cells (Figure 1), as previously reported [14]. Rikkunshito synergistically enhanced the forskolin-induced increase in cAMP levels in a dose-dependent manner, but had little effect on its own.

### Effects of rikkunshito on TH and VIP mRNA levels

**Figure 2:**
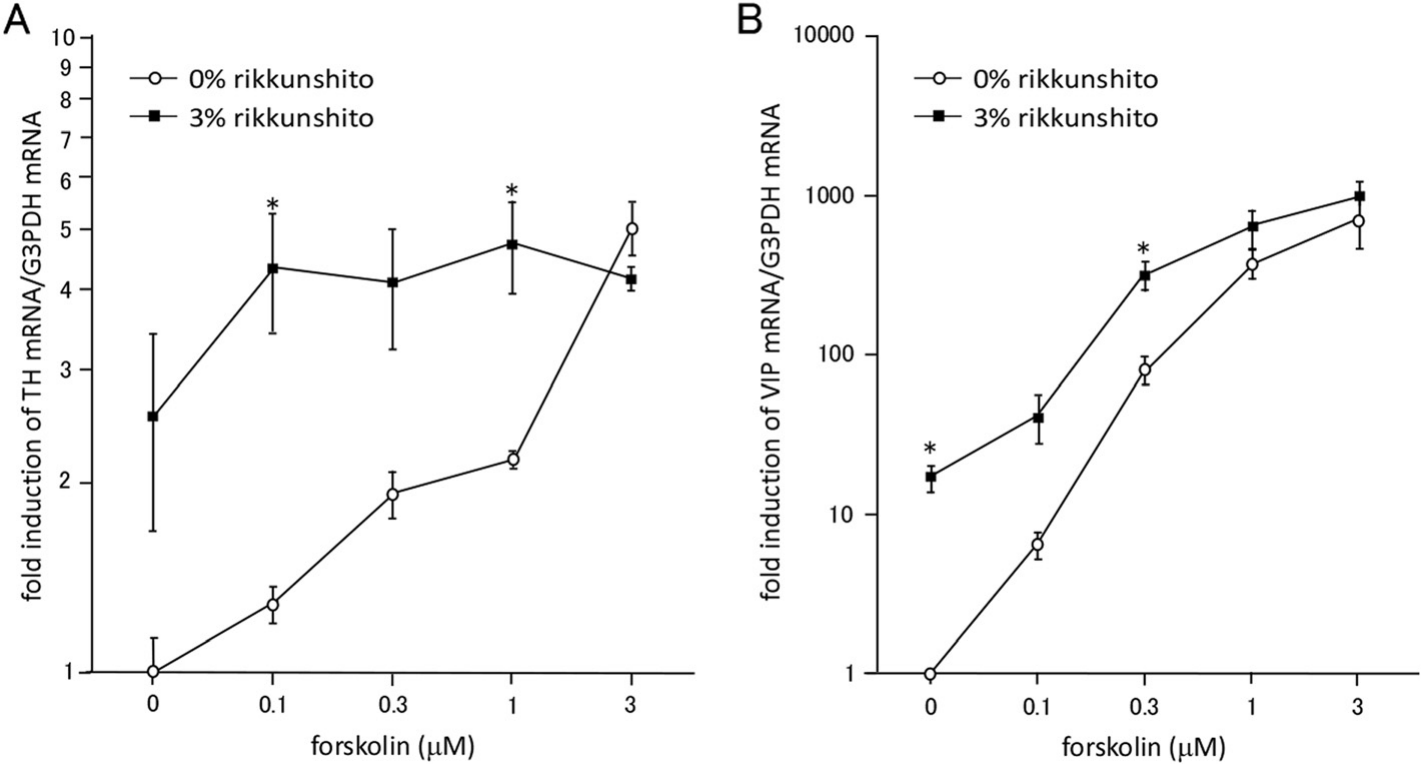
**Rikkunshito increases TH and VIP mRNA levels.** PC12 cells were treated with forskolin alone or in combination with rikkunshito for 6 h. The expression levels of TH mRNA **(A)** and VIP mRNA **(B)** were normalized relative to the expression level of G3PDH mRNA. Data are presented as the mean ± SEM of triplicate independent experiments. *, p < 0.05 vs. 0% rikkunshito at each forskolin concentration tested, two-tailed Student’s *t*-test.

TH and VIP mRNA expression levels were dose-dependently augmented in PC12 cells by forskolin, in agreement with the observations of other investigators [13,15]. Rikkunshito (3%) also increased TH and VIP mRNA expression levels by itself, and further enhanced the expression levels of both mRNAs in the presence of lower concentrations of forskolin (0.1, 0.3, and 1 μM for TH mRNA, Figure 2A; 0.1 and 0.3 μM for VIP mRNA, Figure 2B). However, such further enhancement of gene expression by rikkunshito was not observed when the cells were stimulated with forskolin at higher concentrations.

### Effects of rikkunshito on VIP promoter activity

**Figure 3:**
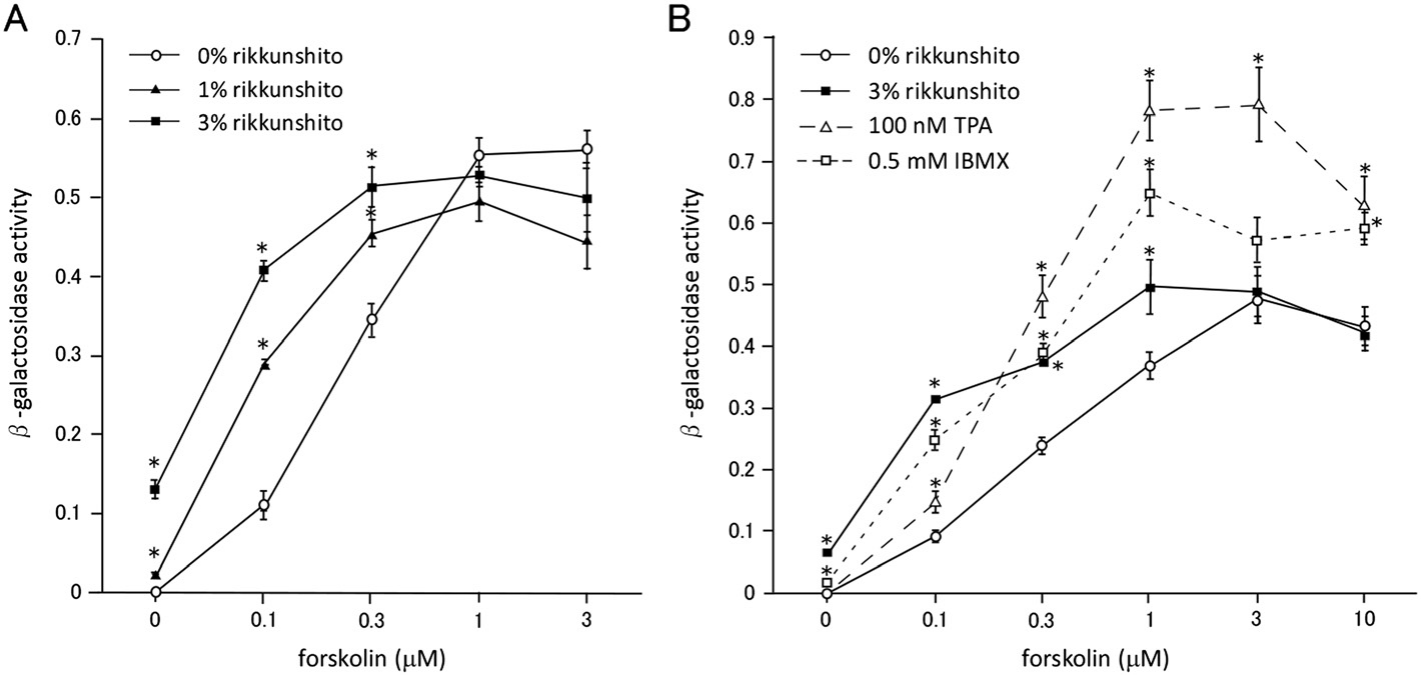
**Rikkunshito stimulates VIP promoter activity in PC12 cells.** PC12 cells were treated with forskolin alone or in combination with rikkunshito **(A)**, or rikkunshito, TPA, or IBMX **(B)** for 6 h. Data are presented as the mean ± SEM of quadruplicate independent experiments. *, p < 0.05 vs. 0% rikkunshito at each forskolin concentration tested, two -tailed Student’s *t*-test.

To confirm that the increased mRNA levels corresponding to the cAMP-responsive TH and VIP genes were due to enhanced transcription, VIP gene promoter activity was measured in PC12 cells expressing the β-galactosidase reporter gene driven by the VIP gene promoter (PC12-VG cells). Forskolin dose-dependently stimulated β-galactosidase activity in these cells, as expected (Figure 3). Rikkunshito also dose-dependently stimulated β-galactosidase activity, and in addition potentiated the actions of forskolin in a dose-dependent manner (Figure 3A). Nonetheless, rikkunshito did not increase the maximum effects observed with forskolin at high concentrations (Figure 3A and B). By contrast, TPA enhanced forskolin-induced β-galactosidase activity over the entire range of forskolin concentrations tested, as previously reported [13], but had no effect by itself. Similar results were obtained in cells treated with forskolin plus IBMX (Figure 3B).

### Effects of rikkunshito on catecholamine release from PC12 cells

**Figure 4:**
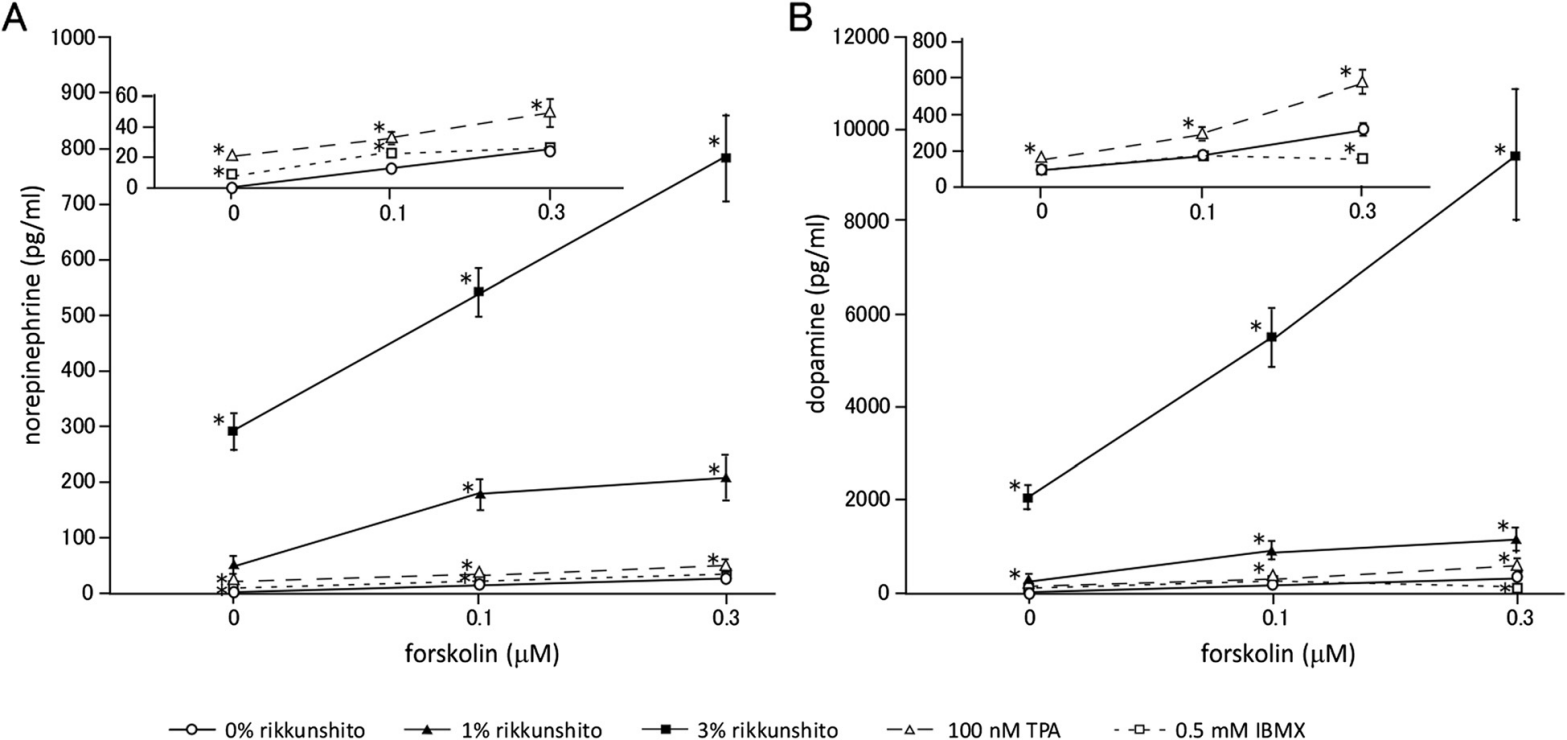
**Rikkunshito promotes catecholamine release from PC12 cells.** Norepinephrine **(A)** and dopamine **(B)** were measured in the culture medium of PC12 cells treated with forskolin alone or in combination with rikkunshito, TPA, or IBMX for 3 h. Data points of 0% rikkunshito, 100 nM TPA, and 0.5 mM IBMX are shown in the insets. Data are presented as the mean ± SEM of triplicate independent experiments. *, p < 0.05 vs. 0% rikkunshito at each forskolin concentration tested, two-tailed Student’s *t*-test.

Unlike normal chromaffin cells, PC12 cells produce norepinephrine and dopamine, but they do not produce detectable amounts of epinephrine [9]. We found that norepinephrine (Figure 4A) and dopamine levels (Figure 4B) were dose-dependently increased in the culture medium of PC12 cells when the cells were stimulated with forskolin, rikkunshito, or a combination of both. The synergistic effects of forskolin (0.1 and 0.3 μM) plus rikkunshito (1 or 3%) are clearly demonstrated in Figure 4, and were considerably greater than those of forskolin plus 100 nM TPA or forskolin plus 0.5 mM IBMX.

## Discussion

This study investigated the effects of rikkunshito on catecholamine release from rat pheochromocytoma PC12 cells. Rikkunshito increased intracellular cAMP levels in combination with forskolin (Figure 1) and enhanced the expression of the TH and VIP genes (Figures 2 and 3), both of which are activated by cAMP at the transcriptional level [13,15]. Importantly, cAMP was previously shown to augment catecholamine release from PC12 cells [16]. We also observed enhanced catecholamine release by rikkunshito (Figure 4), which presumably is mediated at least in part by cAMP. Among eight crude drugs of rikkunshito, ginseng and citrus unshu peel most potently enhanced VIP promoter activity (our unpublished data), suggesting that these drugs may have the compounds responsible for the increase of cAMP. As ginseng contains a substantial amount of adenosine and PC12 cells have adenosine A2A receptor, which activates adenylate cyclase and induces synthesis of cAMP, adenosine may be responsible for the cAMP-inducing effect of rikkunshito.

In agreement with earlier work [13], TPA, a protein kinase C (PKC) activator, increased the maximum actions of high concentrations of forskolin to induce VIP gene promoter activity in PC12 cells (Figure 3). IBMX, a phosphodiesterase inhibitor, also increased the maximum actions of forskolin. By contrast, rikkunshito acted in synergy with lower concentrations of forskolin, but did not enhance the maximum effect of higher concentrations of forskolin. These findings suggest that rikkunshito does not simply modulate PKC and/or phosphodiesterase activities to influence catecholamine synthesis in and/or release from PC12 cells.Also noteworthy is that rikkunshito by itself has little effect on cAMP level (Figure 1), but clearly stimulated TH and VIP gene expression and catecholamine release (Figures 2 and 4). These findings suggest the involvement of a signaling pathway other than those activated by cAMP. Because 100 nM TPA alone and 0.5 mM IBMX alone have only limited effects on VIP promoter activity (Figure 3B) and catecholamine release (Figure 4), other mechanisms than protein kinase C activation and phosphodiesterase inhibition should be operating. A candidate mechanism might be activation of ion channels involved in catecholamine release. Further study is necessary to elucidate the protein kinase A and C-independent effects of rikkunshito.

Previous studies demonstrated that ginseng significantly reduces stress-induced elevations in TH and dopamine β-hydroxylase (DBH) mRNA levels in the rat adrenal gland, and that ginseng total saponin also reduces nicotine-induced TH and DBH gene expression in PC12 cells [12]. Furthermore, glycyrrhetinic acid, one of the main components of glycyrrhiza, inhibits phosphodiesterase activity and increases intracellular cAMP levels in various tissues *in vivo*[17]. While these studies indicate that the components of rikkunshito exhibit distinct biological activities, they do not fully explain the actions of rikkunshito demonstrated in the present study. It is likely that the capacity of rikkunshito to influence cAMP levels and catecholamine metabolism in PC12 cells stems from additive, synergistic, and antagonistic interactions among the diverse constituents of the herbal formulation, rather than from the actions of any single component.

Rikkunshito is employed as an antiemetic agent for the management of a variety of conditions, including chemotherapy-related dyspepsia in cancer patients [7]. Thus, rikkunshito may find utility as part of a regimen to treat cancer cachexia, which is characterized by anorexia, body weight loss, muscle and fat wasting, and biochemical abnormalities related to a state of hyponutrition. Previous work showed that muscle wasting in animal models of cancer cachexia was diminished by β-adrenergic agonists (e.g., formoterol and clenbuterol), with a possible mechanism of action attributed to the inhibition of muscle proteolysis [18,19]. Rikkunshito might therefore be beneficial in ameliorating cachexia-associated muscle wasting, if its *in vitro* actions to augment catecholamine release are recapitulated *in vivo.* Further whole-animal investigations will be required to explore this hypothesis.

## Conclusions

This study demonstrated for the first time that rikkunshito increases cAMP levels and augments catecholamine release from PC12 cells through an as yet undetermined mechanism. Elucidation of the mechanism of action of rikkunshito will foster our understanding of the therapeutic efficacy of this herbal formulation.

## Competing interests

The authors declare that they have no competing interests.

## Authors’ contributions

TT and YU developed the study idea, supervised the study and data interpretation. YN carried out the experiment, drafted the manuscript, and engaged in data acquisition and data interpretation. KT participated in the design of the study. YN and TT were responsible for manuscript editing. All authors read and approved the final manuscript.

## Pre-publication history

The pre-publication history for this paper can be accessed here:

http://www.biomedcentral.com/1472-6882/14/256/prepub

## References

[B1] Araki Y Mukaisho K Fujiyama Y Hattori T Sugihara H The herbal medicine *rikkunshito* exhibits strong and differential adsorption properties for bile salts Exp Ther Med 2012 3 645 649 22969945 10.3892/etm.2012.478 PMC3438557

[B2] Kawahara H Kubota A Hasegawa T Okuyama H Ueno T Ida S Fukuzawa M Effects of rikkunshito on the clinical symptoms and esophageal acid exposure in children with symptomatic gastroesophageal reflux Pediatr Surg Int 2007 23 1001 1005 17668223 10.1007/s00383-007-1986-7

[B3] Okuno S Hirayama K Inoue J Yamasaki K Kawahara R Yokoi T Saeki N Funato T Effects of rikkunshito on the postoperative nausea and vomiting (PONV) after laparoscopic gynecological surgery Masui 2008 57 1502 1509 19108494

[B4] Shiratori M Shoji T Kanazawa M Hongo M Fukudo S Effect of rikkunshito on gastric sensorimotor function under distention Neurogastroenterol Motil 2011 23 323 e156 21175995 10.1111/j.1365-2982.2010.01648.x

[B5] Fujitsuka N Asakawa A Uezono Y Minami K Yamaguchi T Niijima A Yada T Maejima Y Sedbazar U Sakai T Hattori T Kase Y Inui A Potentiation of ghrelin signaling attenuates cancer anorexia-cachexia and prolongs survival Transl Psychiatry 2011 1 e23 22832525 10.1038/tp.2011.25 PMC3309517

[B6] Takeda H Sadakane C Hattori T Katsurada T Ohkawara T Nagai K Asaka M Rikkunshito, an herbal medicine, suppresses cisplatin-induced anorexia in rats via 5-HT2 receptor antagonism Gastroenterology 2008 134 2004 2013 18439428 10.1053/j.gastro.2008.02.078

[B7] Tomono H Ito Y Watanabe T Successful antiemetic treatment of TSUMURA rikkunshi-to extract granules of ethical use in addition to other antiemetic agents in neoadjuvant chemotherapy for an advanced breast cancer patient Jpn J Cancer Chemother 2006 33 1129 1131 16912533

[B8] Yasukawa K Yu SY Kakinuma S Takido M Inhibitory effect of rikkunshi-to, a traditional Chinese herbal prescription, on tumor promotion in two-stage carcinogenesis in mouse skin Biol Pharm Bull 1995 18 730 733 7492991 10.1248/bpb.18.730

[B9] Greene LA Tischler AS Establishment of a noradrenergic clonal line of rat adrenal pheochromocytoma cells which respond to nerve growth factor Proc Natl Acad Sci U S A 1976 73 2424 2428 1065897 10.1073/pnas.73.7.2424 PMC430592

[B10] Kim C-H Koo B-S Kim K-O Kim J-K Chang Y-C Lee I-S *Salviae miltiorrhizae* radix increases dopamine release of rat and pheochromocytoma PC12 cells Phytother Res 2006 20 191 199 16521109 10.1002/ptr.1833

[B11] Watano T Nakazawa K Obama T Mori M Inoue K Fujimori K Takanaka A Non-competitive antagonism by hirsuteine of nicotinic receptor-mediated dopamine release from rat pheochromocytoma cells Japan J Pharmacol 1993 61 351 356 8320880 10.1254/jjp.61.351

[B12] Kim Y Choi E-H Doo M Kim J-Y Kim C-J Kim C-T Kim I-H Anti-stress effects of ginseng via down-regulation of tyrosine hydroxylase (TH) and dopamine β-hydroxylase (DBH) gene expression in immobilization-stressed rats and PC12 cells Nutr Res Pract 2010 4 270 275 20827341 10.4162/nrp.2010.4.4.270 PMC2933443

[B13] Tsukada T Fukushima M Takebe H Nakai Y Vasoactive intestinal peptide gene expression in the rat pheochromocytoma cell line PC12 Mol Cell Endocrinol 1995 107 231 239 7768335 10.1016/0303-7207(94)03448-3

[B14] Block T Kon C Breckenridge BM Mutants of PC12 cells with altered cyclic AMP responses Mol Cell Biol 1984 4 2091 2097 6095039 10.1128/mcb.4.10.2091-2097.1984 PMC369026

[B15] Wessels-Reiker M Haycock JW Howlett AC Strong R Vasoactive intestinal polypeptide induces tyrosine hydroxylase in PC12 cells J Biol Chem 1991 266 9347 9350 1674510

[B16] Baizer L Weiner N Regulation of dopamine release from PC12 pheochromocytoma cell cultures during stimulation with elevated potassium or carbachol J Neurochem 1985 44 495 501 2981284 10.1111/j.1471-4159.1985.tb05441.x

[B17] Amer MS McKinney GR Akcasu A Effect of glycyrrhetinic acid on the cyclic nucleotide system of the rat stomach Biochem Pharmacol 1974 23 3085 3092 4155299 10.1016/0006-2952(74)90593-0

[B18] Busquets S Figueras MT Fuster G Almendro V Moore-Carrasco R Ametller E Argilés JM López-Soriano FJ Anticachectic effects of formoterol: a drug for potential treatment of muscle wasting Cancer Res 2004 64 6725 6731 15374990 10.1158/0008-5472.CAN-04-0425

[B19] Carbó N López-Soriano J Tarragó T González O Llovera M López-Soriano FJ Argilés JM Comparative effects of β_2_-adrenergic agonists on muscle waste associated with tumour growth Cancer Lett 1997 115 113 118 9097986 10.1016/s0304-3835(97)04718-6

